# Menstruation associated hypocalcemic symptoms and serum calcium in patients with idiopathic hypoparathyroidism

**DOI:** 10.1186/1472-6823-14-28

**Published:** 2014-03-21

**Authors:** Soma Saha, Ravinder Goswami

**Affiliations:** 1 Department of Endocrinology and Metabolism, All India Institute of Medical Sciences, New Delhi 110029, India

**Keywords:** Hypoparathyroidism, Hypocalcemic symptoms, Ionized calcium, Menstruation

## Abstract

**Background:**

Some of the patients with idiopathic hypoparathyroidism (IHP) report symptoms of hypocalcemia during menstruation. There is limited data on this observation.

**Methods:**

Twenty six menstruating women with IHP and 26 healthy controls were questioned regarding symptoms suggestive of hypocalcemia during menstruation. Twelve patients and eight controls were asked to prospectively monitor symptoms suggestive of hypocalcemia and premenstrual syndrome (PMS) if any, over two consecutive menstrual cycles. Serum ionized calcium (SiCa^++^), total and albumin adjusted calcium and intact paratharmone (iPTH) were measured at eight points covering menstrual, immediate post-menstrual, mid-cycle and premenstrual phase.

**Results:**

Twelve of the 26 (46.2%) patients with IHP reported hypocalcemic symptoms during menstruation as compared to none of the controls. During prospective monitoring, there was no specific trend of hypocalcemic symptoms with respect to the phase of menstrual cycle. The mean SiCa^++^, serum total and albumin-adjusted calcium, iPTH and inorganic-phosphorus measured over two menstrual cycles were not significantly different in either of the two study groups. None of the subjects had PMS.

**Conclusion:**

Women with IHP do not show any trend of hypocalcemic symptoms or fluctuations in serum calcium over different phases of menstrual cycles. Therefore, patients with hypoparathyroidism linking hypocalcemic symptoms with menstruation should be reassured regarding lack of this association.

## Background

Idiopathic hypoparathyroidism (IHP) is characterized by tetany, tingling, numbness, cataract, convulsion, hypocalcemia, hyperphosphatemia and subnormal serum PTH levels [1]. These patients are managed with daily oral calcium and vitamin D to maintain serum total calcium in the range of 2.0 – 2.13 mmol/l [1]. During past 15 years we have investigated a large cohort of patients with IHP and reported several new clinical manifestations of the disease [2-13]. A subset of the patients complained of hypocalcemic symptoms in the perimenstrual phase. Though several investigators have reported similar clinical observation [14-18], there is only one systematic study and an isolated case report investigating relationship of serum calcium with menstruation in patients with hypoparathyroidism [19,20] and the results were variable. It was also suggested that hypocalcemic symptoms during perimenstrual phase might be a reflection of PMS rather than hypocalcemia [19].

In the present study, we serially monitored SiCa^++^, total calcium and PMS over two menstrual cycles to assess their relationship with hypocalcemic symptoms in women with IHP.

## Methods

Patients were 26 menstruating women with IHP attending endocrine clinics of the All India Institute of Medical Sciences (AIIMS), New Delhi during 2012. Their clinical and biochemical profiles were similar to that described in earlier studies [2-10]. All the patients were asked regarding occurrence of menstruation associated hypocalcemic symptoms such as tetany, peri-oral tingling, numbness and seizures during past six months. Twelve of them were further subjected to serial measurement of biochemical parameters over two consecutive menstrual cycles. The selection criteria for these 12 patients were (a) their willingness to follow up during two successive menstrual cycles as per the study design and (b) sufficient literacy to maintain menstrual diary for recording of PMS and hypocalcemic symptoms. Controls were 26 age matched (± two years) healthy medical and paramedical staff with regular menstrual cycles employed at AIIMS. Eight of them consented for monitoring of biochemical parameters during menstrual cycle as per the study design.

Menstrual history regarding regularity and duration of cycles was recorded for all the subjects. Blood samples were drawn after overnight fast at eight points during two successive cycles (point a – h) as follows: Cycle 1- (a) day 1 or 2; (b) day 3 or 4; (c) 2 days after cessation of menstruation (d) 9–10 days after cessation of menstruation and (e) 1–3 days prior to their expected date of next menstruation. In the next cycle also blood samples were drawn on (f) day 1 or 2; (g) day 3 or 4; and (h) 2 days after cessation of menstruation.

Serum was separated from blood after centrifugation at 1500 × g under cold condition and stored at -20C for measurement of total calcium, phosphorus, albumin and iPTH. SiCa^++^ was measured soon after the blood sampling.

Patients were advised to continue the prescribed dose of calcium and vitamin D, dietary habits, exercise schedule and were provided telephonic contact of the authors for emergency advice. Calcium carbonate (Elder pharmaceutical Ltd, Mumbai) and, 1-α (OH)D (Panacea Pharmaceutical, India) were provided to the non affording subjects to facilitate compliance with the therapy. Facility for travel was also provided for repeated hospital visits during the study.

### Assessment of premenstrual syndrome and hypocalcemic symptoms

The menstrual diary was provided to the study subjects to track symptoms suggestive of premenstrual syndrome (PMS) on daily basis for two consecutive months. The diary included recording of twelve physical and five psychosomatic symptoms of PMS described in the questionnaire available at http://www.womenshealth.gov/publications/our-publications/pms-symptom-tracker.pdf. The questionnaire was translated to the native Hindi language by the authors. Subjects were also asked to record occurrence of hypocalcemic symptoms such as muscle cramps, tetany, tingling and numbness in the limbs in the dairy on daily basis. The diagnosis of PMS was based on presence of (a) one or more ‘affective’ (irritability, depression, angry outbursts, confusion, anxiety and social withdrawal) or ‘somatic’ symptoms (headache, breast tenderness, abdominal bloating and swelling of extremities) occurring during five days before menstrual bleed, (b) relief from these symptoms within 4 days after menstruation with no recurrence until 13^th^ day of the cycle and (c) presence of above pattern of symptoms during at least two consecutive cycles [21].

The study protocol was approved by the ethics committee of All India Institute of Medical Sciences, New Delhi. Written informed consent was obtained from all the subjects.

### Biochemical estimation

Serum total calcium, inorganic phosphorus, albumin and alkaline phosphatase were measured (Modular P 800, Roche/Hitachi, Mannheim, Germany, normal range (NR): 2·0–2·6, 0·8–1·4 mmol/l and 80–240 IU/l, respectively) as described earlier [2], with intra and inter-assay coefficients of variation ranging between 3·5% and 5·0% for both in all the assays. SiCa^++^ was measured by Roche 9180 electrolyte analyzer with inter and intra assay coefficients of variation of 0.6% and 1.3% (Normal values = 1.12 ± 0.05 mmol/l). Serum iPTH was measured by chemiluminscence assay (Elecsys-2010, Roche, Mannheim, Germany, NR 15–65 ng/l) in the endocrine service laboratory.

### Statistical analysis

Data is given as mean and SD. Differences in the variables between patients and controls were assessed by students‘t’ and chi square tests. ANOVA with Bonferroni correction was used to assess significance of difference between means of biochemical parameters at eight study points in the patient and the control groups.

## Results

**Figure 1:**
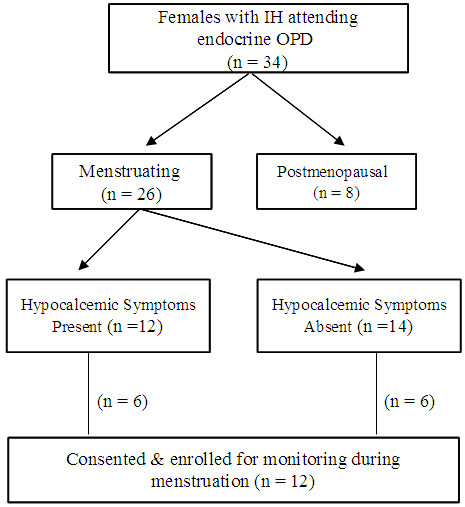
Flow diagram showing patients enrolled in the study.

Figure 1 shows the flow diagram for the study. The duration of menstruation of 26 patients with IHP varied from two to six days. Twelve of them had history of hypocalcemic symptoms namely, numbness, tingling and carpopedal spasm during menstruation. Patients with (n = 12) and without (n =14) menstruation associated hypocalcemic symptoms had comparable mean age (28.0 ± 7.09 yr *vs*. 31.1 ± 9.45 yr, P = 0.35), duration of illness (6.2 ± 4.97 *vs*. 7.4 ± 7.6 yr, P = 0.62), serum total calcium (1.5 ± 0.24 *vs*. 1.4 ± 0.25 mmol/l, P = 0.55), inorganic phosphorus (2.2 ± 0.29 *vs*. 2.2 ± 0.45 mmol/l, P = 0.72) and iPTH (10.2 ± 13.2 *vs.* 11.3 ± 11.96 ng/l, P = 0.62) at diagnosis of illness.

The mean age of 26 controls was similar to that of patients (30.7 ± 6.11 *vs*. 29.4 ± 8.24 yrs, P = 0.53) and none of them had menstruation associated hypocalcemic symptoms. The mean age and BMI of 12 patients and eight controls consenting for prospective monitoring were also comparable (29.3 ± 6.11 *vs*. 25.8 ± 4.43 yrs and 20.5 *±* 2.53 *vs*. 23.2 ± 3.5 kg/m^2^_,_ P = 0.18 and 0.06, respectively). All of them had regular menstrual cycles (28 ± 1 week). The duration of menstruation varied from 2–6 days in patients and 4–8 days in the controls. Premenstrual sample was drawn 1–5 days before next menstruation in patients and 1–6 days before in controls.

### Prospective monitoring during menstrual cycle

Table 1 shows the SiCa^++^ and serum total calcium values during two cycles and their relationship with hypocalcemic symptoms recorded by the patients in their menstrual diary. Hypocalcemic symptoms were recorded by eight patients (Table 1, bold digits) but by none of the controls. Most of the patients with hypocalcemic symptoms in the first cycle had these symptoms in the second cycle also. The mean SiCa^++^, serum total calcium (unadjusted and albumin adjusted), iPTH and inorganic phosphorus levels at all the study points were comparable in patients with IHP (Table 2). Similarly, the mean values of these parameters were comparable at all points within the control group.

**Table 1:** Serum ionized and total calcium in patients with idiopathic hypoparathyroidism and controls during menstruation and their relationship with hypocalcemic symptoms

	**Patients with idiopathic hypoparathyroidism**								**Healthy women**							
**Study points**	**Menstrual cycle 1**					**Menstrual cycle 2**			**Menstrual cycle 1**					**Menstrual cycle 2**		
	**a**	**b**	**c**	**d**	**e**	**f**	**g**	**h**	**a**	**b**	**c**	**d**	**e**	**f**	**g**	**h**
**Serum ionized Ca (mmol/l)**																
1*	1.04	**1.12**	1.11	0.92	**0.92**	**0.90**	**0.91**	**0.93**	1.09	1.20	1.16	1.13	1.19	1.14	1.13	1.12
2	0.78	0.77	0.88	0.81	0.91	0.84	0.88	0.91	1.19	1.14	1.13	1.17	1.19	1.13	1.12	1.16
3*	0.85	-	0.82	0.97	0.86	0.86	-	**0.85**	1.12	1.17	1.17	1.16	1.11	1.15	1.17	1.09
4	0.96	0.94	1.01	1.06	0.98	0.92	-	0.90	1.12	1.12	1.15	1.17	-	-	1.07	1.13
5*	**0.91**	**0.83**	**0.82**	**1.15**		**0.82**	**0.86**	**0.91**	1.13	1.18	1.17	1.14	1.17	1.10	1.13	1.12
6	1.04	1.05	1.05	1.04	1.08	1.09	1.05	1.03	1.12	1.13	1.17	1.18	1.09	1.13	1.12	1.15
7	1.04	0.96	0.92	1.01	1.01	**1.04**	-	0.91	1.17	1.14	1.17	1.16	1.11	1.09	1.12	1.19
8	1.13	1.16	1.13	1.16	1.14	1.11	1.09	1.12	1.16	1.17	1.14	1.15	1.16	1.18	1.15	1.21
9*	**1.07**	**1.06**	**1.00**	**1.04**	**0.95**	**0.89**	**0.93**	**0.86**	-	-	-	-	-	-	-	-
10*	**0.84**	**0.89**	**0.93**	**0.90**	-	-	-	-	-	-	-	-	-	-	-	-
11	**0.83**	**0.94**	**0.93**	**0.77**	**0.85**	**0.79**	**0.77**	**0.77**	-	-	-	-	-	-	-	-
12*	**0.79**	**-**	**0.84**	**0.82**	**0.94**	**0.92**	**-**	**0.85**	-	-	-	-	-	-	-	-
**Serum total Ca (mmol/l)**																
1*	2.05	2.00	1.98	2.13	1.93	1.88	2.00	-	2.43	2.18	2.30	2.30	2.38	2.30	2.30	2.25
2	2.05	1.90	1.78	1.52	2.08	1.68	1.80	1.95	2.38	2.40	2.33	2.30	2.33	2.55	2.45	2.35
3*	1.70		1.83	1.70	1.78	1.78	-	1.73	2.20	2.28		2.53	2.48	2.43	2.38	2.35
4	2.05	2.05	1.98	2.25	1.98	1.90	-	2.40	2.33	2.30	2.30	2.25	-	-	2.25	2.33
5*	1.88	1.70	1.75	2.50		1.65	1.70	1.95	2.23	2.40	2.30	2.25	2.50	2.35	2.30	2.35
6	1.93	2.28	2.15	2.15	2.03	2.20	2.10	2.10	2.33	2.48	2.38	1.83	2.30	2.40	2.63	2.30
7	1.88	1.80	2.03	2.08	1.95	2.13	-	1.83	2.40	2.35	2.30	2.25	2.23	2.28	2.23	2.58
8	2.25	1.98	2.00	2.08	1.90	2.03	2.08	2.18	2.55	2.40	2.48	2.48	-	-	-	2.38
9*	2.15	2.13	2.10	2.15	1.88	1.85	1.95	1.78	-	-	-	-	-	-	-	-
10*	1.70	1.73	1.90	1.80	-	-	-	-	-	-	-	-	-	-	-	-
11	1.73	1.85	1.85	1.63	1.68	1.48	1.70	1.55	-	-	-	-	-	-	-	-
12*	1.63		1.78	1.90	1.85	1.88	-	1.73	-	-	-	-	-	-	-	-

Cells with bold digits indicates point during menstrual cycles when patients recorded hypocalcemic symptoms. *Patients with past history of hypocalcemic symptoms during menstruations.

**Table 2:** Various parameters (Mean ± SD) at different study points in the menstrual cycles in patients with idiopathic hypoparathyroidism and controls

**Parameters**	**Menstrual cycle 1 (study points)**					**Menstrual cycle 2 (study points)**			P*
	**a**	**b**	**c**	**d**	**e**	**f**	**g**	**h**	
**Idiopathic hypoparathyroidism**									
Serum ionized Ca (mmol/L)	0.9 ± 0.12	1.0 ± 0.12	1.0 ± 0.11	1.0 ± 0.13	1.0 ± 0.09	0.9 ± 0.11	0.9 ± 0.11	0.9 ± 0.10	0.89
Serum total Ca (mmol/L)	1.9 ± 0.20	1.9 ± 0.18	1.9 ± 0.13	2.0 ± 0.28	1.9 ± 0.12	1.9 ± 0.21	1.9 ± 0.17	1.9 ± 0.25	0.89
Albumin adjusted total Ca (mmol/l) (mmol/L)	1.9 ± 0.19	1.9 ± 0.19	1.9 ± 0.13	1.9 ± 0.24	1.8 ± 0.11	1.8 ± 0.17	1.9 ± 0.16	1.9 ± 0.22	0.85
Inorganic PO_4_ (mmol/L)	1.7 ± 0.29	1.7 ± 0.36	1.8 ± 0.32	1.8 ± 0.28	1.6 ± 0.31	1.8 ± 0.35	1.8 ± 0.49	1.7 ± 0.30	0.90
IPTH (ng/L)	14.9 ± 19.99	16.6 ± 24.43	17.5 ± 23.36	14.2 ± 18.62	15.3 ± 18.86	16.1 ± 20.52	19.9 ± 23.96	14.9 ± 19.99	0.99
**Healthy controls**									
Serum ionized Ca (mmol/L)	1.1 ± 0.03	1.2 ± 0.03	1.2 ± 0.02	1.2 ± 0.02	1.14 ± 0.04	1.1 ± 0.03	1.1 ± 0.03	1.1 ± 0.04	0.25
Seum total Ca (mmol/L)	2.4 ± 0.11	2.4 ± 0.09	2.3 ± 0.07	2.3 ± 0.21	2.35 ± 0.11	2.4 ± 0.10	2.4 ± 0.14	2.4 ± 0.10	0.82
Albumin adjusted total Ca (mmol/l)	2.3 ± 0.09	2.3 ± 0.09	2.3 ± 0.07	2.2 ± 0.24	2.33 ± 0.09	2.4 ± 0.13	2.4 ± 0.16	2.3 ± 0.11	0.46
Inorganic PO_4_ (mmol/L)	1.2 ± 0.16	1.2 ± 0.13	1.2 ± 0.12	1.3 ± 0.15	1.18 ± 0.18	1.2 ± 0.10	1.2 ± 0.14	1.3 ± 0.17	0.63
iPTH (ng/L)	48.0 ± 28.02	42.1 ± 23.80	53.9 ± 29.86	52.0 ± 39.74	34.2 ± 14.20	49.9 ± 36.20	47.3 ± 33.62	41.8 ± 31.31	0.92

P values: ANOVA with Bonferoni correction.

### Premenstrual syndrome (PMS)

None of the patients or controls had symptoms suggestive of PMS. Hypocalcemic symptoms during monitoring did not conform to the pattern of PMS.

## Discussion

McCullagh and Kearns for the first time in 1935 reported exacerbation of hypocalcemic symptoms during menstruation in patients with post surgical hypoparathyroidism [14]. Subsequently, similar observations were made in IHP and ‘autoimmune polyendocrinopathy-candidiasis-ectodermal dystrophy’ associated hypoparathyroidism [16,20]. However, there are only two reports systematically assessing changes in serum calcium in patients with hypoparathyroidism during menstrual cycle [19,20]. Graham *et al*. observed no major fluctuation in serum total calcium during menstruation in six patients with post surgical hypoparathyroidism. The authors suggested PMS as the possible reason for the hypocalcemic symptoms during menstruation in hypoparathyroidism [19]. On the contrary, Mallette LE reported a fall in SiCa^++^ and increased muscle stiffness and during menstruation in a patient with IHP [20].

The present study systematically assessed a larger cohort of women with IHP and controls for hypocalcemic symptoms during different phases of menstrual cycle along with measurement of SiCa^++^, serum total and albumin adjusted calcium. Though approximately half of the women with IHP experienced hypocalcemic symptoms during menstruation, close monitoring revealed no specific trend of these symptoms over different phases of menstrual cycle and symptoms occurred irrespective of the days of cycle.

Furthermore there were no significant differences in the mean SiCa^++^ or total calcium at eight points of measurements during menstrual cycle in IHP. Similar results for SiCa^++^, and total calcium were observed in healthy controls. The results of the present study are similar to that of Graham *et al.*[19], suggesting absence of significant change in serum total and ionized calcium during menstruation in patients with IHP. However, contrary to their suggestion, hypocalcemic symptoms observed by the patients in the present study could not be attributed to PMS.

The pathophysiology of menstruation associated hypocalcemic symptoms in IHP is not clear. However, these symptoms could be subjective and might be a reflection of neuropsychiatric manifestations. In a recent study from our center, patients with IHP demonstrated a high prevalence of somatic concern (25.8%), anxiety (47%), tension (55%) and depression (40.3%) [12].

## Conclusion

Women with IHP on therapy do not show any association of hypocalcemic symptoms or significant fluctuations in serum ionized or total calcium with menstruation. Therefore, patients with hypoparathyroidism linking hypocalcemic symptoms with menstruation should be reassured regarding lack of this association.

## Abbreviations

IHP: Idiopathic hypoparathyroidism; PMS: Premenstrual syndrome; SiCa++: Serum ionized calcium; iPTH: Intact paratharmone.

## Competing interests

The authors declare that they have no competing interests.

## Authors’ contributions

Both the authors contributed in the study design, collection of the data and writing of the manuscript. SS has measured the serum ionized calcium. RG has been managing the cohort of the patients with IHP in the endocrine clinic since 1998. Both authors read and approved the final manuscript.

## Pre-publication history

The pre-publication history for this paper can be accessed here:

http://www.biomedcentral.com/1472-6823/14/28/prepub
